# Twenty‐first‐century chemical odyssey: fuels versus commodities and cell factories versus chemical plants

**DOI:** 10.1111/1751-7915.13379

**Published:** 2019-02-22

**Authors:** Juan L. Ramos, Estrella Duque

**Affiliations:** ^1^ CSIC – Estación Experimental del Zaidín c/Profesor Albareda 1 18008 Granada Spain

## Abstract

The harmful effects of pollution from the massive and widespread use of fossil fuels have led various organizations and governments to search for alternative energy sources. To address this, a new energy bioprocess is being developed that utilizes non‐edible lignocellulose – the only sustainable source of organic carbon in nature. In this mini‐review, we consider the potential use of synthetic biology to develop new‐to‐nature pathways for the biosynthesis of chemicals that are currently synthesized using classical industrial approaches. The number of industrial processes based on starch or lignocellulose is still very modest. Advances in the area require the development of more efficient approaches to deconstruct plant materials, better exploitation of the catalytic potential of prokaryotes and lower eukaryotes and the identification of new and useful genes for product synthesis. Further research and progress is urgently needed in order for government and industry to achieve the major milestone of transitioning 30% of the total industry to renewable sources by 2050.

## Introduction

Kyoto and Paris protocols echo the demands of society to combat global climate change and call for the use of clean, green and renewable transportation fuels as well as sustainable avenues to produce chemicals. These series of measures are motivated by the devastating environmental problems associated with fuels and chemical industries based on petroleum (Isikgor and Becer, 2015). The alternative to the current petrochemical industry is a new ‘chemistry’ based on plant materials, the only sustainable source of organic carbon on earth; this new chemistry could move us towards net zero emissions (Ragauskas *et al*., 2006; Somerville *et al*., 2010; Taarning *et al*., 2011; Zhou *et al*., 2011; Isikgor and Becer, 2015; Ramos *et al*., 2016). Currently, the major source of plant‐derived materials for producing many chemicals is starch, the hydrolysis of which yields mainly glucose. As such, the use of starch for production of biofuel and biochemical commodities is controversial because of the overlap with the food chain. This controversy led to a paradigm shift at the end of the last century when scientists began working to replace petrol as a source of raw material by using non‐edible plant biomass (lignocellulose). Although this idea emerged about two decades ago, it has still not become a reality because the intrinsic recalcitrance of the lignocellulose material makes it an expensive source of sugars. The price for sugar production from grain is around 16 US cent lb^−1^, while production costs for sugars from corn stover are in the order of 30 cent lb^−1^ and at least 45 cent lb^−1^ if the lignocellulose is derived from trees or organic matter from urban waste. At the industrial level, the difference in sugar price according to source is paramount, and as such, efforts have been directed at shortening the price gap between sugars from starch and sugars from lignocellulose and urban wastes. This has driven the discovery of more efficient ways to derive sugars from biomass (Valdivia *et al*., 2016; Roback and Balcerek, 2018). This article explores the production of biofuels and biochemicals using microbial cell factories and some of the current strategies based on sugars derived from starch and lignocellulosic substrates. At present, the chemical industry produces thousands of chemicals, many of them in very large quantities, using fossil oils. Academic and industrial organic chemistry laboratories synthesize new chemicals every year, although only a few of them find industrial use. The current use of fossil hydrocarbons as a source for gasoline, diesel, jet fuel and other fuels is several orders of magnitude higher than the use of them to produce industrial chemical commodities; severely limiting the availability of petrol as a raw material for synthesis of valuable chemicals. One could envisage a dispute of fuels versus chemical commodities in coming years, similar to the one seen previously for food versus fuel regarding the use of grain as a source for ethanol at the end of the 20th century. The current number of ongoing large‐scale biocatalytic processes that use plant material is very low, although there are signs of growth and the Organisation for Economic Co‐operation and Development (OECD) and other agencies are aiming to transition 30% of the total industry to renewable sources by 2050 In fact, Frank (2010) reported that about two‐thirds of in the world's most‐used chemicals could be synthesized from renewable raw materials, rather than from oil; it was estimated that the size of the market for biochemical production from sugars derived from plant polysaccharides is in the order of US $1 trillion. An absolute requirement to consolidate any ‘Cell Factory’, as a source of biochemicals, is that the biologically produced chemicals that reach the market should be able to compete economically with the same product made through chemical synthesis. If the result is not economically feasible, the only way the biologically produced chemical can reach the market is through transitory subsidies, i.e., on saving for instance, on carbon footprint, which needs to be confirmed through complete life‐cycle assessments. The production cost of a biochemical is influenced by a number of factors, among which is the cost of the raw materials used, the production yield and the purity and percentage of recovery of the product(s) from the reaction mixture. The question is, will we be able to produce the full panoply of chemicals from plant materials? The answer will come from (1) current knowledge and the exploitation of advances in the deconstruction of starch and lignocellulose into component sugars, (2) the immense catalytic potential of prokaryotes and lower eukaryotes that can be utilized for synthesis of value‐added chemicals and (3) new synthetic biology approaches that combine, like ‘Lego pieces’, genes from different origins that when judiciously recruited and expressed, give rise to new pathways. Briefly rounding out these three points: (i) deconstruction of starch by amylases and glucoamylases is a consolidated field which already has yields of glucose from starch above 93%. However, deconstruction of cellulose and hemicellulose requires enzymatic cocktails that contain a set of enzymes that need to function coordinately in series. The current enzymatic cocktails converting the polysaccharide in lignocellulose into their constitutive monosaccharides barley reach yields of 80%, although the field of cellulases/hemicellulases is advancing fast (Álvarez *et al*., 2016); (ii) Timmis *et al*. (2017) highlighted, that numerically, the main source of genes is the microbial world, which is not only the most predominant form of life on the planet but also the most relevant source of metabolic diversity in the tree of life. This in part derives from the fact that microbes inhabit all environments on Earth, even the most hostile ones that are incompatible with most forms of life. This has led to a set of biochemical activities that delineate the barriers between the biosphere and the geosphere and which are not present in temperate environments. Modern exploration of the Earth's microbial biosphere is based on metagenomic approaches that can identify genes from microbes that we are not able to cultivate in the laboratory. This, along with the advances in screening using miniaturized devices (e.g. microfluidics), is unveiling new enzymes and reactions, many not believed to exist a few years ago and regarding point 3), we should say that having new enzymes in hand is not sufficient to make new pathways, because they often need to be integrated with cell metabolism. Metabolic engineering seeks to use our current knowledge of living systems to design and create new strains that can function as biofactories to produce value‐added products (Clomburg *et al*., 2017). Early advances in the use of synthetic pathways were focused on the construction of catabolic pathways, via horizontal and vertical expansion, to degrade recalcitrant and xenobiotic compounds (Ramos and Timmis, 1987). In the last decade, synthetic biology (SynBio) has developed novel tools and strategies for the rational design of pathways that are new to nature (Schuster *et al*., 2000; Stephanopoulos, 2012; Nikel *et al*., 2016). A key focus of a number of research programmes is the design of genetic constructs containing the information necessary to synthesize the product(s) of interest from specific substrate(s).

Despite this effervescence in the knowledge of molecular microbial ecology and bioinformatics (King *et al*., 2016), the number of biochemicals (referred to here as a chemical produced by a living organism) made in recent years at pilot‐industrial scale is very modest. A question arises – can biochemicals made from renewable sources replace chemicals (here the term is used to refer to molecules made in the chemical realm), particularly when large amounts are required such as in the world of commodities?

## First‐generation (1G) biofuels and bioproducts

United Nations (2016) recommendations led to significant effort being directed towards producing new non‐contaminant fuels to replace a significant fraction of gasoline, diesel and jet fuel with biofuels in transportation. To be a viable alternative, a biofuel should fulfil a series of recommendations dictated by Hill *et al*. (2006), namely, the biofuel should (i) provide a net energy gain, (ii) have environmental benefits, (iii) be economically competitive and (iv) be produced in large amounts without reduction in food supplies. The same statements regarding environmental benefits, economic feasibility and production in large amounts without affection of the food chain should also be applied to production of biochemicals aimed to replace a compound made by chemical synthesis. Biofuels and biochemicals that fulfil these requirements reduce CO_2_ emissions, and in the case of biofuels, help to reduce particulate emissions by motor vehicles (Fargioni *et al*., 2008) providing an immediate impact on air quality and life expectancy (Kim *et al*., 2015).

Biofuel production from cereal grain (USA and Europe) and sugarcane (Brazil) is a mature industry. The main biofuels derived from starch are ethanol, butanol, isobutanol and microbiological biodiesel. These biofuels are known as first‐generation (1G) or 1G ethanol, 1G butanol, 1G isobutanol of 1G microdiesel.

From a quantitative point of view, the most relevant biofuel (and biochemical) in the world is ethanol; nearly 98% of the total ethanol used in many different industries (energy, solvent, pharmaceutical, etc.) is of biological origin and is produced via hexose fermentation by yeast (global production being ~25 billion gallons per year). This model shows that biological systems have the potential to produce chemical commodities. Nearly 80% of the total global production of ethanol comes from the USA and Brazil, where it is most often blended with gasoline for transportation. It is estimated that 1G ethanol production in the USA and Brazil serves to replace ~8% of the total petrol barrels imported by these countries. This reduces dependence on external oil suppliers and, in addition, saves petroleum that can be kept as a valuable ‘reserve’ for the future.

In the ethanol production process from, i.e., corn grain, the grain is triturated (ground to a fine powder) and mixed with hot water to make a mash. Upon heating and treatment with amylases and glucoamylases, the starch is transformed into glucose as the main product. This glucose is then efficiently fermented by yeasts and > 90% of the sugars converted into ethanol. However, making just ethanol from corn grain is not economically viable, unless a wide series of subproducts are collected and sold; i.e., CO_2_ from fermentation is harvested, liquefied and used for carbonated drinks and medical gases. Then, the dry distilled grain (DGG) – which is the solids that are left upon fermentation and which contain undigested corn components and yeast – is sold as feed for ruminants.

Modern ethanol production plants are highly efficient and have similar yields worldwide; however, the economic returns of 1G ethanol plants are strongly influenced by the price of grain and the natural gas price – the main source of energy in these plants. In the USA, the price of natural gas is more favourable than in Europe and this makes US plants more profitable.

A number of studies within the biofuel field are positioning butanol as a better blending component than ethanol in gasoline. Green (2011) highlighted two relevant benefits for butanol as a biofuel: namely, that butanol has higher fuel density than ethanol; concomitantly it can be blended with gasoline at a rate of 1.6:1; and that butanol is highly compatible with existing petroleum pipe systems so there are transportation benefits from existing facilities. It has also been proposed that butanol and ethanol can be blended together to make a better fuel blending agent (Elfasakhany, 2015), with the optimal ratio being 18% butanol and 12% ethanol (Brandon and Eizeke, 2015).

Biobutanol is mainly produced by fermentation in the acetone–butanol–ethanol (ABE) process. This is a classical industrial fermentation process carried out by a number of strains of the genus *Clostridium* with an A:B:E ratio of 3:6:1 (Green, 2011). The set of strains used in ABE fermentation is very broad and includes strains from different species, although based on performance and robustness, the strains industrially used are *C. acetobutylicum* CACE 979, *C. beijerinckii* NCIMB 8052 and *C. beijerinckii* BA101. In this process, the commercial solvent titres peak at about 20 g l^−1^ from 55 to 60 g l^−1^ of substrate; this gives solvent yields of ~0.35 g/g sugar, which is close to 90% of the theoretical yield (Qureshi and Blaschek, 2008; Jimenez‐Bonilla and Wang, 2018). Therefore, ABE fermentation from a biological point of view is a well‐optimized process.

Butanol is the preferred solvent of the three made in the ABE process since it attracts the highest price in the chemical market, and for this reason, attempts to increase the butanol ratio have been made. One of the most successful examples is that reported by Lee's group in Korea, which showed that an increase in butanol can be achieved by engineering the traditional ABE pathway to convert sugars into butanol through the so‐called hot channel; the modified strain had an acetone:butanol:ethanol ratio of 3:8:1. (Jang *et al*., 2012). Still with this improvement, it was found that the solvent‐tolerance ability of *Clostridium* limited the economic viability of the process.

The clostridia ABE fermentation process is relatively simple, and new fermentation plants can be constructed through retrofitting of 1G starch ethanol plants. Techno‐economical analysis has shown that the retrofit model provides an attractive option to expand renewable 1‐butanol production in the USA and Brazil because it allows for an increased profit margin (close to 30%). The most valuable retrofitting approach is based on the use batch‐fed fermentation technology combined with continuous solvent extraction to avoid the toxic effects of butanol (> 20 g l^−1^) (Jimenez‐Bonilla and Wang, 2018). This technology allows a more efficient use of sugars present in the feed stream and a constant production of solvents.

As mentioned above, the ABE process suffers due to the production of less interesting industrial products (acetone and ethanol), and for this reason, attempts to produce butanol that is free of these solvents have been explored by using other pathways. For instance, alternative pathways for production of butanol were searched by Liao's group, which showed that butanol can be made from 2‐ketovalerate, a central metabolite in *E. coli* (Atsumi *et al*., 2008). However, the process was restricted by solvent‐sensitivity and the limited activity of the Lactococcus lactis keto‐acid decarboxylase used. The same pathway was explored in solvent‐tolerant aerobic *Pseudomonas* strains; however, the strain metabolizes butanol, and different metabolic blocks were needed to achieve significant butanol productivity (Cuenca *et al*., 2016).

Atsumi *et al*. (2008) reported titres of 22 g l^−1^ of isobutanol with engineered *Escherichia coli* strains that produced this alcohol using diversion of central metabolites in the valine biosynthetic pathway, and Baez *et al*. (2011) reported that the engineered *E. coli* (JCL260) strain produced more than 50 g l^−1^ in 72 h if isobutanol was removed from the bioreactor using gas stripping.

LS9, a spin‐off company from the University of California, has developed a platform technology that exploits microbial fatty acid biosynthesis pathways to produce a number of so‐called dropin fuels. Using synthetic biology, LS9 has constructed different *E. coli* strains that allow conversion of carbohydrates to biodiesels, namely, a fatty acid methyl ester (biodiesel ASTM 6751) and an alkane (ASTM D975) (Bokinsky *et al*., 2011). To achieve this, first an *fad*E *E. coli* MG1655 knock‐out mutant that accumulated fatty acid ethyl ester was constructed. Later the production was increased by expressing the *fad*D, *alf*A, *pdc*,* adh*B, *tes*A and *alf*A genes from the *lac*UV5 promoter. The LS9 processes are said to be unique in that all of the chemical conversions from carbohydrates to finished fuels are catalysed in the cell, with the finished product secreted. In fact, the fuel seems to form an immiscible light organic phase that is nontoxic to *E. coli* and is easily recovered from the broth through centrifugation (Rude, 2011, 2014; Groban *et al*., 2015). LS9 claims that these simple processes enable the production of diesel from scalable renewable resources at a price competitive with petroleum.

## Bioproducts from starch

From an economic point of view, ethanol, butanol and microbial biodiesel are highly volatile products because their selling prices are influenced by the price of grain, the price of natural gas used as the energy source in the production plants and petrol prices due to its main use as blending for gasoline. As an alternative to biofuel production, a number of companies are interested in producing biochemicals from sugars, either through retrofitting of existing ethanol plants or through building new specific plants.

The ethanol industry that uses starch is very well consolidated, production costs are very well documented and simple equations can be derived to determine the economic feasibility of making a bioproduct from starch using for instance a retrofitted ethanol plant or to estimate CAPEX for building a new one. For these calculations, what is required is to establish the theoretical maximal yield of the biotransformation and to fix the expected industrial yield of the process and the potential costs for the recovery of the products from the production stream. This together with estimated capital investments allows one to define the returns of the project. Alper and colleagues suggested that key central metabolites (pyruvate, citric acid, tyrosine, aspartate and acetyl‐CoA) are critical metabolic nodes for biosynthesis of specialty and commodity chemicals (Markham and Alper, 2015; Cordova and Alper, 2016). Our own analysis identified a series of products, itaconic acid, succinic acid, isoprene, acrylic acid, lactic acid and mid‐chain alcohols such as octanol, derived from the nodes that can be economically profitable. These compounds in turn can be used as building blocks to manufacture a wide variety of polymers that are currently produced from expensive and price volatile petroleum feedstocks (Isikgor and Becer, 2015). A number of companies are exploring the production of some of these organic compounds, i.e. succinic acid, citric acid, isoprene and lactic acid, from starch.

The market potential for succinic acid is estimated to be $7.6 billion per year. In the conventional petrochemical process, succinic acid is made by oxidation of benzene or butane to produce maleic anhydride, which is then converted to succinic acid through hydrolysis. Several drawbacks have been identified in the chemical process, such as the toxic catalysts which are used, that nearly 50% of the carbon in the raw materials is lost as CO_2_ and that a series of undesired products is present which makes downstream purification a requirement (Isikgor and Becer, 2015). The microbial production of succinic acid is now becoming a mature technology (Wang *et al*., 2013). Succinic acid is produced by a number of different microorganisms from different substrates including sugars. Current production levels with recombinant *E. coli* strains are above 10 g l^−1^ (Wang *et al*., 2013), and although bio‐succinic acid costs are still higher than the petroleum‐based counterpart, it is still of interest because: (i) bio‐succinic acid is produced free of many other chemicals present in the chemically synthesized product, (ii) that bio‐succinic acid is produced at room temperature and ambient pressure in contrast with the high temperature, and (iii) that the biological process is favourable with respect to the loss of C from the raw materials. Furthermore, an additional positive factor for bio‐succinic acid is that several companies have reported that renewable polymers such as polyester polymers can be made from biologically produced succinic acid and 1,4‐butanediol (Adkins *et al*., 2012).

Bacterial routes to produce lactic acid account for > 90% of all lactic acid production; for this, strains of *Lactobacillus acidophilus* and *Streptococcus thermophilus* are used. Generally, starch is used as a feedstock and transformation yields greater than 90% of the theoretical are obtained. The production of enantiomer pure l‐lactic and d‐lactic acid depends on the bacterial strain used. Acrylic acid cannot be made by living organisms, but it can be produced from lactic acid through chemo‐selective full conversion of lactic acid into acrylic acid over a calcium pyrophosphate catalyst at 375°C (Isikgor and Becer, 2015).

Isoprene is used in a variety of applications, including the production of synthetic rubber. A number of plants produce isoprene from dimethylallyl pyrophosphate through the action of isoprene synthase. Yang *et al*. (2012) developed a process for the production of isoprene in *E. coli*, in which the set of genes for mevalonate biosynthesis from *Enterobacter faecalis* (those encoding HMG‐CoA synthase, acetyl‐CoA acetyltransferase and HMG‐CoA reductase which convert acetyl‐CoA into MVA) were coexpressed with the isoprene synthase (IspS) from *Populus alba*. The corresponding genes were synthesized *in vitro* with codon usage optimized for *E. coli* and then provided with promoters so that they enabled the construction and rapid characterization of metabolically engineered strains to produce isoprene. The best recombinant strain produced up to 6 g l^−1^ isoprene (Liu *et al*., 2014). Green metrics analysis indicated that biological‐based isoprene production, though slightly costlier than the petrochemical one, is of economic and environmental interest (Morais *et al*., 2015). Relevant efforts to improve *E. coli*,* Pseudomonas* and a number of yeast to produce a new set of value‐added chemicals have and are still being made (Calero and Nikel, 2018; Liu *et al*., 2013).

## Second‐generation (2G) biofuels and bioproducts

**Figure 1:**
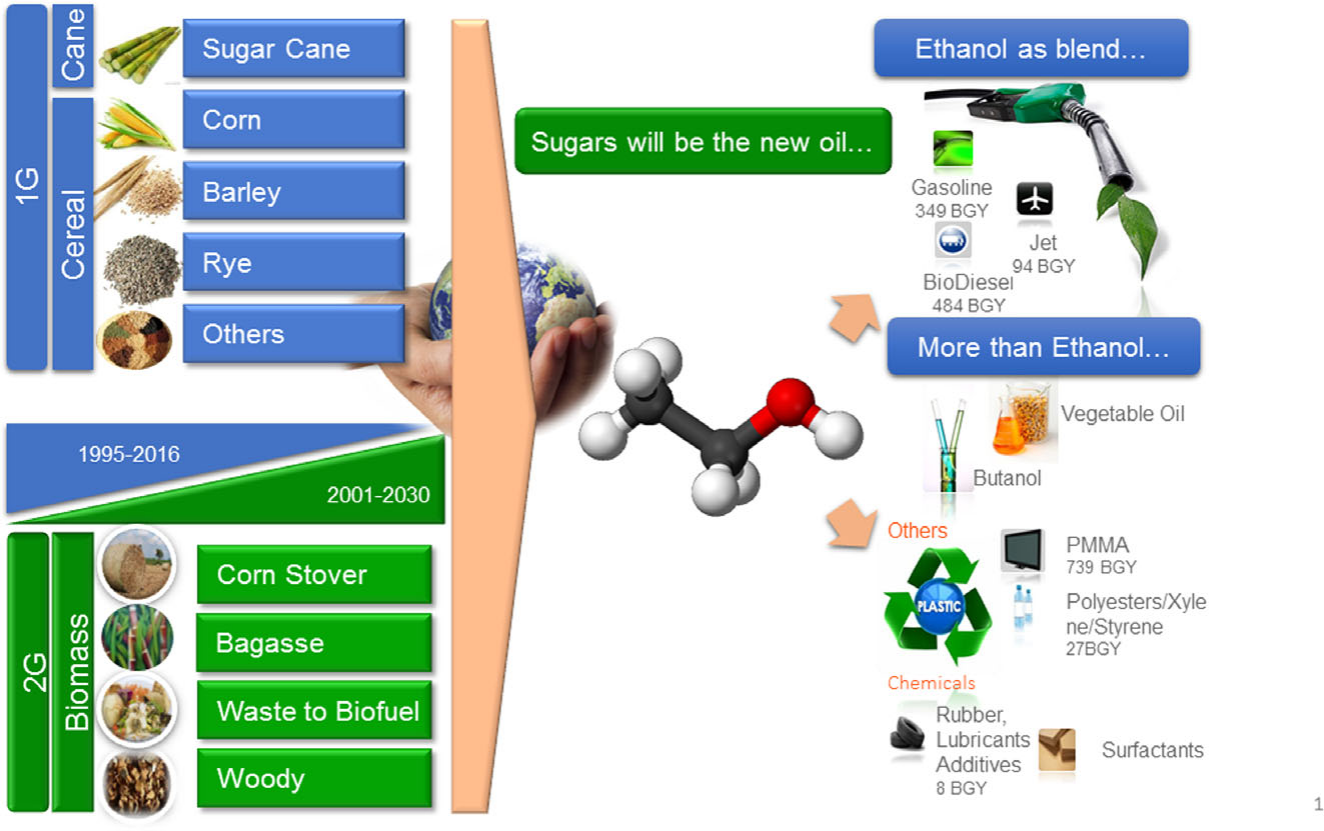
Proposed synthesis of bioproducts from plant materials as envisioned by Abengoa.

As mentioned above, at the end of the last century a fierce fight broke out regarding food vs fuel; this instigated the search for new sources of glucose and other sugars to use as raw chemicals for the synthesis of biomolecules. In fact, one of the grand challenges of the biofuel/bioproduct industry is to integrate their production into current Circular Economy trends (Fig. 1). Since half of the organic carbon in the biosphere is present in the form of cellulose, the conversion of cellulose into valuable chemicals has paramount importance (Ragauskas *et al*., 2006; Somerville *et al*., 2010; Zhou *et al*., 2011; Isikgor and Becer, 2015;). Hemicellulose follows cellulose as the second most abundant polymer and has an amorphous structure made of several heteropolymers such as xylan, galactomannan, glucuronoxylan, arabinoxylan and others.

Although the original aim of using lignocellulose was for the production of biofuels, a new trend of interest has been directed at generating value‐added chemicals. Products derived from lignocellulose are called second‐generation (2G) biofuels and (2G) bioproducts. A number of studies support that biofuels and biochemicals produced from agricultural residues (i.e. corn stover, bagasse, sugar cane straw, olive tree branches and intensive greenhouse crop residues) are sustainable and environmentally friendly (Valdivia *et al*., 2016; Ko and Lee, 2018). However, lignocellulose has evolved to resist degradation and its recalcitrance derives from crystalline cellulose, the encapsulation of cellulose by the lignin–hemicellulose matrix and the inherent hydrophobicity of lignin (Barakat *et al*., 2013). As such, the use of cellulose and hemicellulose as a source of sugars is complex. In fact, the production of 2G biofuels and 2G biochemicals requires three major steps in series, namely, (i) a physicochemical pre‐treatment to release cellulose and hemicellulose from lignin in lignocellulosic materials; (ii) a set of enzymatic cocktails to breakdown cellulose/hemicellulose polymers into their constituents – monosaccharides – a process called saccharification and (iii) the subsequent fermentation of the sugars to target compounds (Taherzadeh and Karimi, 2007; Álvarez *et al*., 2016; Oh *et al*., 2018).

The first step is pre‐treatment aims to break the natural binding characteristics of lignocellulose by modifying the supramolecular structure of the cellulose–hemicellulose–lignin matrix to make the polysaccharides bioavailable. A number of pre‐treatment technologies are known, including chemical, physical and biological processes. Some of these technologies have already been commercialized and are well known, whereas others are still at laboratory scale. For instance, steam explosion is a well‐known technology which consists of heating the biomass in water under pressure followed by a sudden decompression of the reaction vessel. As a result of the violent decompression, the structure of lignocellulose is disrupted and the fibres are opened up, leaving sugar polymers more accessible to the subsequent enzymatic hydrolysis (Klinke *et al*., 2004). The main drawbacks of steam explosion are related to the mildness of the process, which limits the effectiveness of the pre‐treatment and demands the use of very high enzyme loads in the saccharification step (Brownell *et al*., 1986; Yang and Wyman, 2008).

A combination of steam explosion with the use of dilute aqueous solutions of inorganic acid (HCl, H_2_SO_4_) or base (ammonia) results in good depolymerization and release of hemicellulose and cellulose. This kind of pre‐treatment requires high capital investment because the use of acid or base causes corrosion and special reactor metallurgy is needed. Lignin removal is another key step in the development of the biofuel industry, and in the above two pre‐treatments, lignin is maintained until the distillation phase.

Several research groups and companies (reviewed by Yoo *et al*., 2017) have developed an extraction method for highly pure cellulose and hemicellulose from lignocellulose based on the use of ionic liquids (IL) (Socha *et al*., 2004), which are able to dissolve lignocellulose under mild conditions, resulting in more accessible cellulose and recovery of lignin in the raw material (Tadesse and Luque, 2011). At present, the current IL prices make them non‐competitive at the industrial scale (Ding *et al*., 2016).

The second step in the 2G technology process is enzymatic hydrolysis to convert the polysaccharides into monosaccharides using enzymatic cocktails. Several fungi (*Myceliopthora, Aspergillus, Pichia* and others) are used to produce enzymatic cocktails that consist of a broad set of enzymes that are secreted into the growth broth. Hydrolysis of cellulose and hemicellulose requires the synergistic action of a set of enzymes to break them down. Endo‐β‐1,4‐glucanases, exo‐β‐1,4‐glucanases (i.e. cellobiohydrolases) and β‐1,4‐glucosidases are necessary for the efficient breakdown of cellulose (Álvarez *et al*., 2016). The degradation of hemicellulose is more complex and requires the concerted action of backbone depolymerizing enzymes (endoxylanases and β‐xylosidases), as well as accessory enzymes to hydrolyse side‐chains on the xylan backbone (α‐l‐arabinofuranosidases, acetyl xylan esterases, feruloyl esterases and α‐glucuronidases) (Álvarez *et al*., 2016). A general limiting factor in lignocellulose hydrolysis is the attack of crystalline cellulose/hemicellulose; the recalcitrance of this fraction can be overcome using lytic polysaccharide monooxygenases (PMOs), a set of copper‐dependent enzymes that act on crystalline polysaccharides and use oxygen as a substrate (Álvarez *et al*., 2016; Vaaje‐Kolstad *et al*., 2017).

Currently, the fungi used in production are mainly genetically engineered strains that have been improved to express cellulases, hemicellulases and other accessory enzymes with the aim of enabling the release of at least 80% of the sugars present in celluloses and hemicelluloses as monosaccharides. Industrial production fungi secrete 50–100 g of protein per kg of broth. This very high production trend is the result of the combination of expression of target genes from strong promoters, optimized secretion capacities of the producing fungi and optimized growth parameters that allow the high protein production levels. In nature, the breakdown of lignocellulosic material is achieved through the combined action of fungi and bacteria, although production of cellulases by bacteria is at least one order of magnitude lower than fungi, following from this concept, we have recently reported the design of a symbiotic enzyme cocktail that combines fungal and bacterial enzymes to digest lignocellulose (Duque *et al*., 2018). This cocktail is made of a basic fungal cocktail supplemented with hemicellulases isolated from ruminal bacteria, the ‘symbiotic’ cocktail increased the total amount of sugars released from lignocellulosic material up to 20%, making the production of 2G sugars more efficient and viable as raw material for the synthesis of biofuels and bioproducts.

Enzymatic hydrolysis represents ~25–30% of the operational costs in current 2G processes, whereas in 1G, the enzyme cost is below 3% (Álvarez *et al*., 2016). Therefore, reducing the enzymatic cocktail cost contribution is a must for the viability of 2G technology. Significant attention has been paid by academic research groups and industry to improve the production and performance of 2G enzymes; the aim being to establish what is called ‘the minimal 2G cocktail’ – this is the lowest amount of all enzymes required to reach the industrial goal of producing sugars at prices similar to those of the 1G technology. Programmes to enhance cocktail effectiveness include removal of non‐productive enzymes from the cocktail, replacement of some of the wild‐type enzymes by recombinant versions that are catalytically superior to the original (Farinas *et al*., 2001; Mate and Alcalde, 2017) or by heterologous enzymes that perform better. Current research to improve 2G cocktails is aimed at finding thermostable enzymes capable of performing for longer periods at temperatures above to 60°C and which are resistant to proteases.

## Bioproducts from 2G sugars

In contrast with starch, the hydrolysis of which yields glucose, hydrolysis of cellulose and hemicelluloses yields a mixture of sugars, whose proportion vary with the plant material. Glucose and xylose are the most abundant; in the case of corn stover, glucose represents approximately 70% in total, while xylose and arabinose account for approximately 20% and 3% respectively. The effective biotransformation of these sugars to ethanol or value‐added chemicals requires strategies that allow fermentation of all of these C6 and C5 sugars. Successful fermentation of 2G sugars to alcohols such as ethanol, butanol, alkanes, succinic acid and other bioproducts has been described; we briefly illustrate these processes below.

Yeasts that are utilized in 1G ethanol production are unable to ferment the C5 sugars present in the enzymatic hydrolysate of cellulose/hemicellulose. The processing of C5 sugars is a must in 2G technology because they account for nearly 25% of the total sugars and as mentioned above pre‐treatment and enzymatic cocktails are expensive. As such, a number of companies and laboratories have developed very efficient recombinant yeasts that harbour native xylose‐assimilating routes from bacteria, allowing co‐fermentation of glucose and xylose (Ho *et al*., 1998). These yeasts simultaneously ferment glucose and xylose to ethanol, with more than 96% glucose and more than 90% of xylose converted into ethanol (Álvarez *et al*., 2016; Ko and Lee, 2018; Sharma *et al*., 2018). While this is true with herbaceous lignocellulose material, when the raw material comes from wood, a series of inhibitors is generated during the pre‐treatment that make the fermentation processes less efficient (Heer and Sauer, 2008; Tomás‐Pejó and Olsson, 2015). A recent SynBiol development in the field is the SCRaMbLe technology that generates diverse pools of yeast mutants from which strains that have valuable industrial characteristics can be isolated. For example, Luo *et al*. (2018a) used SCRaMbLe to accelerate the isolation of yeast strains that are tolerant to various stress factors, such as ethanol, heat and acetic acid. SCRaMbLe is expected to allow important developments in the synthesis of biochemicals. We developed yeast strains that fermented arabinose to ethanol; the approach we took was to provide C6 yeast with an arabinose transporter from *Spathaspora passalidarum* plus a series of ara genes from *Peidococcus pentosus*. The initial assemblage of genes did not result in growth on arabinose (Caballero and Ramos, 2017), but yeasts were submitted to a series of evolution assays to adapt them to ferment arabinose, and eventually was isolated a strain capable of fermenting more than 90% of the arabinose to ethanol (Caballero and Ramos, 2017).

Some of the solvent producing *Clostridia* described above for the 1G butanol technology bare the set of enzymes needed to ferment C5 sugars derived from cellulose feedstocks, and in fact *C. saccharobutylicum* P262, *C. beijerinckii* P260 and *C. beijerinckii* BA101 use a broad range of pentose sugars (Qureshi and Blaschek, 2008). These strains showed good fermentation performance with corn fibre (Qureshi *et al*., 2008; Luo *et al*., 2018b), wheat straw and dried distillers grains. Furthermore, these strains tolerate the growth inhibitors formed during the pre‐treatment of hard‐wood (eucalyptus plants), and interestingly, the furans (formed from sugar degradation) were found to stimulate butanol production (Zheng *et al*., 2015). Reported solvent yields and titres are considered acceptable (Green, 2011) but demonstration of the technical and economic feasibility of butanol produced from cellulose at scale is required.

Recombinant yeasts and *E. coli* that produce succinic acid from 2G sugars have been constructed (reviewed by Liu *et al*., 2013). In the case of yeast, two steps were needed: in the first, microbes co‐fermented both hexoses and pentoses derived from digested lignocellulose were created as described above by recruiting xylose degradation pathways. The developed yeast strains operated at low pH (in the range between 4.5 and 5) which avoided contamination in the fermentation process. To cost effectively produce succinic acid, Yuzbashev *et al*. (2010) showed that if one of the subunits of succinate dehydrogenase is deleted the strain will not grow on sugars aerobically, but it can grow on glycerol and ferment sugars to succinic acid at rates about 2.5 g l^−1^ h^−1^, reaching up to 45 g l^−1^ in buffered culture media. In the case of the *E. coli* production using corn, hydrolysates were in the range of 10 g l^−1^. Therefore, production of 2G succinic acid by biological means appears to be feasible.

The examples above clearly show that it is possible to use 2G sugars as substrates for production of biofuels and value‐added chemicals. The new set of synthetic biology tools should now allow us to accelerate the construction of strains ‘Bio Factories’ that contain very efficient pathways and potent detoxification mechanisms.

## Conclusions and perspectives

The main driver behind the push towards both 1G and 2G biofuel and biochemical production is market need. This push requires continuous collaboration between technologists, the production enterprises and the final users so that a chain of ‘green’ chemistry develops from the bottom up. Advances in SynBiol provide a number of possibilities for the design of appropriate circuits to produce biofuels and biochemicals with high added‐value. We have seen an impressive development in the set of tools that allow the rapid construction and testing of the ‘new‐to‐nature’ circuits. Industry and academia have to look for win–win agreements to advance the field so they can give rise to and consolidate the ‘new’ sugar‐based chemistry. Although the focus of this article is on biological synthesis of chemicals, it is important to mention that this is not the only ‘green’ approach, and that organic chemistry approaches also represent another win–win for industry and academia. We should also mention other drivers for green biofuel and biochemical production, i.e., that substrates are available worldwide, that ‘green’ chemistry/fuels help to secure country's energy supply and reduce its dependence on external oil supplies. In addition, 1G and 2G technologies support rural areas through technology deployment and creation of knowledge‐based jobs, and that these technologies help to mitigate greenhouse gas emissions that are noxious for the environment, animals and humans – promoting a low carbon and sustainable economy. We predict that sugars, although very basic molecules, will replace oil as a starting material from which a myriad of chemicals will be made. This will be brought about by the judicious use of synthetic biology and molecular biology approaches in the bio‐sector and development of new reactions in the organic chemistry arena. In the bio‐sector, there is no single universal microorganism that can be used in production, clearly there are different microbial platforms that can be used to produce a diverse list of chemicals under both oxic and anoxic conditions; we aim to address this issue in a future opinion article.

## Conflict of interest

None declared.
